# “Health First” Instead of “Safety First”

**Published:** 1914-08

**Authors:** M. M. Carrick

**Affiliations:** Dallas, Texas


### EDITORIAL DEPARTMENT.

MRS- F. E. DANIEL, Publisher and Managing Editor.
DR. R. H. L. BIBB, Associate Editor.

CONTRIBUTING EDITORS:

Dr. H. Sheridan Baketel, New York, editor *Medical Times;* Dr. M. H. Boerner, Austin, Texas; Dr. G. Henri Bogart, Paris, Ill.; Dr. M. M. Carrick, Dallas, Texas; Dr. Edward H. Carey, Dean Medical Department, Baylor University, Dallas, Texas; Dr. W. L. Crosthwaite, Waco, Texas; Dr. T. D. Crothers, Hartford, Conn.; Dr. H. W. Cummings, Hearne, Texas; Dr. Oscar Dowling, New Orleans, President Louisiana State Board of Health; Havelock Ellis, M. D., London, Eng.; Dr. May Agnes Hopkins, Dallas, Texas; Dr. A. W. Fly, Galveston, Texas; Dr. G. B. Foscue, Waco, Texas; Dr. E. S. Goodhue, Holualoa, Kona, Hawaii; Dr. M. B. Grace, Seguin, Texas; Dr. Joe Gilbert, Austin, Texas; Dr. Charles W. Hughes, St. Louis, editor *Alienist and Neurologist;* Dr. James G. Kiernan, Chicago; Dr. George H. Lee, Galveston, Texas; Dr. John T. Moore, Houston, Texas; Dr. Frank Paschai, San Antonio, Texas; Dr. John Preston, Austin, Texas, Superintendent State Lunatic Asylum; Dr. Bacon Saunders, Fort Worth, Texas; Dr. Allen J. Smith, Philadelphia, Medical Department, University of Pennsylvania; Dr. J. T. Scott, Austin, Texas; Dr. Ralph Steiner, Austin, Texas, President Texas State Board of Health; Dr. John S. Turner, Dallas, Texas; Dr. Charles Venable, San Antonio, Texas.

### "Health First" Instead of "Safety First."

BY M. M. CARRICK, M. D., DALLAS, TEXAS.

A celebrated German physician recently startled the German Empire by making the statement that more lives were lost annually from preventable diseases than there were soldiers killed in the Franco-Prussian war. Everybody gasped. It seemed incredible.

There is nothing remarkable about this statement after all, for there is not a country in the world that could not make an equally startling one, America included.

When we pause to consider that over 400,000 people are killed or maimed annually by accidents in the United States alone, we are appalled. Yet according to Dr. Josiah Strong and other eminent sociologists these figures are not exaggerated; but how many of us realize that more people are lost annually from preventable diseases than are killed by accidents? The layman interested in figures can secure plenty of them from the United States government statistician in proof of this statement.

In view of these facts, with which we physicians have long been cognizant, is it not time that we should make an effort to replace the "Safety First" sign with one more to the point. "Health First" should be the sign to greet us on every billboard, fence and public building.

We all admit that the "Safety First" movement was a timely one, and it should have the earnest co-operation of the medical profession, as the need of it is great, owing to the demands of this industrial age for speed, regardless of human life and safety.

Industrial leaders have been too absorbed in competitive measures to put much value on human life—it has been useful to them like any other piece of machinery that could be replaced, if necessary. Speed has been insisted upon in everything until the safety movement was launched. This ultimately brought about co-operation between the employer and employe.

Preventive medicine is the most useful field for the medical profession. On it depends the health of the individual, the home, and the State. Our success in preventing disease depends upon the use made of this knowledge. If every city, town and village started a "Health First" campaign, we would soon have better public sanitation, better food, more intelligence care of the child in the home, better ordered play, long childhood and wide open gates of the senses, all making for a better childhood and better men and women.

Why not start to raising funds to placard every conspicuous place with "Health First" signs, and see if we cannot bring the millennium of health about by talking it, living it, working for it until the public is made to realize that we as physicians are actually engaged in a humanitarian work without any thought of personal gain or emolument.

"Health First!"

